# Evaluation of the Effectiveness of Proprioceptive Training According to Radiological Stages in Patients with Knee Osteoarthritis

**DOI:** 10.3390/medicina61030546

**Published:** 2025-03-20

**Authors:** Sibel Gayretli Atan, Esra Pehlivan, Sinan Bağçacı

**Affiliations:** 1 Orthopedic Prosthesis Orthosis Department, Harran Health Services Vocational School, Sanliurfa 63300, Türkiye; 2 Department of Physiotherapy and Rehabilitation, Hamidiye Health Sciences Faculty, Health Sciences University, İstanbul 34668, Türkiye; esra.pehlivan@sbu.edu.tr; 3 Physical Medicine and Rehabilitation Department, Konya Medicana Hospital, Konya 42060, Türkiye; sinanbagcaci@gmail.com

**Keywords:** osteoarthritis, knee, proprioception, strengthening

## Abstract

*Background and Objectives*: The aim of the study was to compare the effectiveness of proprioceptive studies according to radiological stages in patients with knee osteoarthritis and to determine at which stage of the disease it should be added to the rehabilitation program. *Materials and Methods*: This study is a prospective clinical trial. The study was registered with ClinicalTrials.gov (name of the registry: Evaluation of the Effectiveness of Proprioceptive Training According to Radiological Stages in Patients with Knee Osteoarthritis; trial registration number: NCT06150170; date of registration: 21 November 2023). The patients were divided into two groups, which were Grade 1–2 (Group 1) and Grade 3–4 (Group 2) knee osteoarthritis. Both groups underwent a strengthening plus proprioception exercise 3 times a week for 4 weeks. Our primary scale was the Western Ontario and McMaster Universities Arthritis (WOMAC) scale. The secondary outcome measures were pain intensity level, proprioception, range of motion, muscle strength, physical performance, physical activity, quality of life and patient satisfaction. All evaluations were performed twice, before treatment and after 4 weeks of treatment. *Conclusions*: After treatment, there were significant improvements in pain, range of motion, proprioception, muscle strength, functionality, physical performance and quality of life in both groups (*p* < 0.05). There was no significant difference between the total WOMAC scores among groups after treatment (*p* = 0.086). There was more improvement in hip external rotation range of motion in Group 1 (*p* = 0.022). No significant difference was found in other secondary outcomes (*p* > 0.05). As a result of this study, we found that proprioceptive training was effective on pain, joint position sense, range of motion, muscle strength, functionality, physical performance and quality of life in patients with knee osteoarthritis in all radiological stages. However, there was no difference between the groups, except for hip external rotation angles.

## 1. Introduction

Osteoarthritis (OA) is among the most common causes of disability worldwide, and according to the global disability report, more than 527 million people are reported to be affected by OA [1]. Since the knee joint is a weight-bearing joint, it is one of the joints where OA is most common, and the prevalence of knee OA is 13% in women and 10% in men in individuals aged 60 years and older worldwide [2]. Osteoarthritis (OA) is a chronic, degenerative, progressive disease affecting all joints and is characterized by cartilage loss, osteophyte formation, synovial inflammation and problems in bone remodeling [3]. These structural damages cause the collapse of cartilage and subchondral bone, resulting in pain, tenderness and dysfunction in the joint [4]. In the development of the disease, local factors, such as trauma, occupational factors (e.g., jobs that require kneeling and squatting, heavy lifting, etc.), anatomical alignment and general factors, such as age, female gender, vitamin D deficiency and family history, play a role [5,6]. The most prominent symptom of the disease is pain that occurs due to activity and decreases with rest [7]. Other common symptoms are limitation in joint range of motion, morning stiffness, crepitation, joint instability, edema, muscle weakness, fatigue and psychological problems due to pain [8]. The first preferred method in the treatment of the disease is conservative treatment. Conservative treatment consists of patient education, activity modification, weight control, physiotherapy and rehabilitation, pharmacological treatments (non-steroidal anti-inflammatory drugs (NSAIDs), acetaminophen, COX-2 inhibitors), non-pharmacological treatments and injections into the joint [6]. Exercises are strongly recommended by the guidelines in the conservative treatment of knee OA [6,9]. It is known that quadriceps muscle weakness can cause knee OA [10]. A systematic review has indicated that resistive exercises reduce pain and improve functionality in knee OA [11]. In addition, a systematic review reported that the inclusion of hip exercises in the strengthening program will positively affect the treatment in terms of knee biomechanics [12]. Proprioception is defined as the conscious or unconscious perception of the position and movement of an extremity or a joint in space. It also plays a role in the regulation of joint position awareness, kinesthesia and resistance sensation. When healthy individuals of the same age and individuals diagnosed with knee OA are compared, proprioception is worse in patients with knee OA [13]. Inadequate proprioception may cause OA pathogenesis and progression of the disease [14]. Therefore, resistive exercises alone are not sufficient to improve proprioception in the treatment of the disease. It is stated in the literature that proprioception training is necessary to improve proprioception [15]. The exercises used in the content of proprioceptive studies are agility exercises, balance exercises, coordination exercises, plyometric exercises, strength sensation training and repositioning exercises [16]. In a systematic review, there are studies that generally examine the effectiveness of proprioceptive exercises applied in addition to strengthening exercises or only proprioceptive exercises in patients with knee OA at the same radiological level [17]. However, there is no study in the literature comparing the effectiveness of proprioceptive exercises applied in addition to strengthening exercises in patients with knee OA at different radiological levels. Our study evaluates the effectiveness of proprioceptive exercises applied in addition to strengthening exercises separately in OA at different radiological levels. It also compares the effectiveness of proprioceptive studies at different levels. Thus, it shows at which stage of the disease we can add proprioceptive training to the rehabilitation program. We assumed that proprioceptive studies would be superior to Group 2 (3–4) in terms of function, which is the primary outcome measure. We also assumed that Group 1 would be more effective in the secondary outcome measures of pain, proprioception, ROM, muscle strength, physical performance, physical activity, quality of life and patient satisfaction. This study investigates in which radiological stage of the disease proprioception exercises applied in addition to strengthening exercises are more effective on these parameters.

## 2. Materials and Methods

### 2.1. Study Protocol

This clinical study was designed as a prospective, clinical study. This study was performed at Konya Medicana Hospital. The research protocol was approved by the Research Ethics Committee of KTO Karatay University Ethics Board in Konya, Türkiye (institutional review board approval no: 2024-72751; approval date: 27 October 2023). The study was conducted in accordance with the Declaration of Helsinki. Informed consent was provided by all patients prior to their enrollment in the study. The trial was registered at ClinicalTrials.gov (name of the registry: Evaluation of the Effectiveness of Proprioceptive Training According to Radiological Stages in Patients with Knee Osteoarthritis; trial registration number: NCT06150170; date of registration: 21 November 2023).

### 2.2. Participants

Participants were recruited from Konya Medicana Hospital from November 2023 to April 2024. Patients were treated at the Physiotherapy and Rehabilitation Department of Konya Medicana Hospital. The patients were diagnosed by a physical therapy and rehabilitation specialist (S.B.), who is an expert in knee diseases. Patients who were diagnosed with knee osteoarthritis according to the American College of Rheumatology (ACR) criteria between the ages of 35 and 70 and who had Kellgren–Lawrence (KL) stage 1–2–3–4 on radiological examination were included in the study. Patients with active synovitis, those using antidepressants, those who had intra-articular steroid injections in the last 6 months, those with neurological diseases, those who had lower extremity surgery, those with vestibular problems, those who had severe speech, vision and hearing problems and those who had health problems that prevented exercise were not included in the study.

### 2.3. Randomization and Blinding

Group 1 (Grade 1–2) and Group 2 (Grade 3–4) patients were included in the study randomly. Randomization was performed with a computerized system technique.

Blinding: Patients were evaluated by E.P., an expert physiotherapist in the field. SGA treatment was started by an expert physiotherapist in the field. Patients did not have any information about the content of the treatment applied to other patients.

### 2.4. Outcome Measures

The demographic information of the patients participating in the study was recorded using the case assessment form before treatment.

The functional status, pain, range of motion and proprioception, muscle strength, physical performance, physical activity levels and quality of life of the patients were evaluated. In addition, patient satisfaction after treatment was evaluated.

The primary scale in our study was WOMAC. The Western Ontario and McMaster Universities Arthritis (WOMAC) scale was used to assess functional status. There is a negative correlation between the total score and the patient’s functionality [18]. The level of pain felt at rest/activity/night was measured using the “Visual Analog Scale” (VAS) [19]. Pressure pain threshold (PPT) is defined as the lowest pressure value applied to create pain [20]. A digital algometer (J-Tech Algometer) was used to measure the PPT value. While patients were in a supine position, pressure was applied to the midpoint of the knee and 1 cm medial to the heel using a moss 1 cm^2^ probe at a 90° angle to the skin using a 1 cm cap. Patients were asked to say “stop” when they first felt pain. Low values indicate increased sensitivity and pain. A 10 s rest was given between measurements. The measurements were repeated 3 times, and the average was recorded as the result. A digital goniometer (Baseline Evaluation Instrument^®^, Fabrication Enterprises, Inc., Po Box 1500, White Plains, NY, USA) was used to assess joint position sense (JPS) [21]. Knee flexion angles of 75° and 60° were used to assess proprioception. The patient was asked to bring his/her leg from 90° to 75° knee flexion and from 90° to 60° knee flexion. After holding this angle for 5 s, the patient was asked to learn this angle. After a 10 s break, the patient was asked to find these angles again with his/her eyes closed. The difference between the angle taught to the patient and the angle applied by the patient was recorded. This difference was determined as the error score. Each measurement was repeated 3 times, and the average was recorded. A digital goniometer was used to assess the range of motion (ROM) of the lower extremity. Hip flexion, external and internal rotation, knee flexion and extension, foot dorsiflexion and plantar flexion range of motion were measured with a digital goniometer [21]. Lovett’s manual muscle test was used to evaluate muscle strength. The strength of the muscles performing hip flexion, abduction, adduction and extension, knee flexion and extension and foot dorsiflexion and plantar flexion was evaluated [22].

Timed Up and Go Test (TUG) was used to assess physical-function-based performance [23].

The short version of the International Physical Activity Questionnaire (IPAQ) was used to assess physical activity [24]. The Short Form-36 questionnaire (SF-36) was used to assess health-related quality of life. The scale consists of 8 subcategories and a total of 36 questions. Higher scores indicate increased quality of life [25]. In addition to these evaluations, the Global Range of Scale (GRS) was used to assess patient satisfaction after 5 weeks of treatment [26].

#### 2.4.1. Follow-Up

All evaluations were performed before treatment (first assessment) and after 4 weeks in the rehabilitation program (second assessment).

#### 2.4.2. Interventions

**Figure 1:**
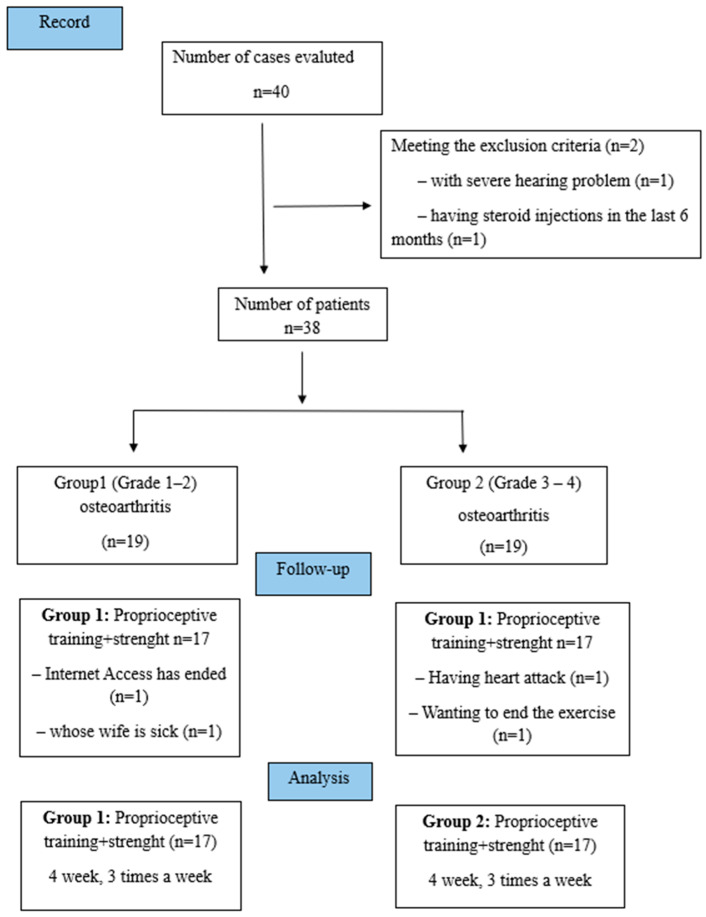
Flow diagram of the study.

Patients were divided into two groups: Group 1 (Grade 1–2 Knee OA) and Group 2 (Grade 3–4 Knee OA) (Figure 1). A rehabilitation program consisting of proprioceptive exercises in addition to strengthening exercises was created for both groups according to the clinical presentations of the patients. All patients in both groups received treatment via telerehabilitation for 4 weeks, 3 days a week.

### 2.5. Rehabilitation Program (Group 1 and Group 2)

The exercise program applied to both groups according to the clinical conditions of the evaluated patients was created from the literature [13,27]. All exercises were progressively performed by patients weekly according to the progression suggested by the literature (specified in Appendix A) and the condition of the patient. The exercises were performed by a physiotherapist. All patients underwent a total of 12 sessions of a telerehabilitation exercise program, 3 sessions per week for 4 weeks. The number, duration and difficulty of the exercises were increased according to tolerance. This exercise program, created from the literature, included proprioception exercises, balance and coordination exercises and strengthening exercises [13,26,27].

### 2.6. Proprioception Exercises

Heel Walk: The patient walks forward on a flat surface on their heels.

Toe Walk: The patient walks forward on their toes.

Sideways Knitting Walk: Step to the side with the right foot, bring left foot behind right, step to the side with right, bring left in front of right; repeat for the prescribed number of steps; change the leading foot and repeat in the opposite direction.

Sideways Step: Stand with feet together, step to the side with the leading foot, bring the trailing foot back to the leading foot; repeat for the prescribed number of steps, then repeat in the opposite direction.

Cross Walk: Walk forward bringing each foot across the midline of the body.

Semi Tandem Walk: Walk heel-to-toe with the heel landing just in front of and medial to the great toe of the opposite foot.

Tandem Walk: Advanced version of the above; heel lands directly in front of the opposite foot.

High Knee Walk: Walk forward while flexing the hip about 90 degrees.

Wedding Walk: Step forward and slightly to one side with the leading foot, bring the trailing foot together with the leading foot; alternate the leading foot.

Backward Wedding Walk: As above, stepping backward [27].

All walks started with 15 steps in the first week and progressed to 30 steps in the last week.

Balance exercises consisted of weight transfer exercises on one leg forward, sideways and backward. The exercises started with eyes open for 10 s and progressed with eyes closed, and the duration increased in the following weeks.

Coordination exercises consisted of bilateral knee flexion–extension exercises with the patient sitting in a 90o flexed knee position and exercises comprising lifting of the heel of one foot from the medial malleolus to the medial condyle of the tibia and lowering it again, starting from the medial malleolus of the leg. The exercises started with 10 repetitions with eyes open and progressed with eyes closed, and the number increased in the following weeks.

Strengthening exercises consisted of open kinetic chain and closed kinetic chain exercises. A 5 min warm-up light jog was performed before the exercises. Hip abduction, adduction, flexion and extension exercises, knee flexion and extension exercises showed progression without weight in the first week, and the resistance was increased by 0.5 kg each week. Squat exercises started with 8 repetitions and progressed to 12 repetitions in the last week. The progression of all exercises according to weeks is shown in Appendix A.

### 2.7. Data Analysis

The sample size was evaluated using “G*Power software (version 3.1.9.2)”. Calculations were performed to detect a difference with 80% power (β level 20%) and 0.05 significance level (α level 0.05) at 95% confidence interval. In a previous study, G*Power was calculated using WOMAC, one of the primary outcome measures in the previous study [28]. According to the calculated measurement, a total sample size of 34 patients was created, with at least 17 patients per group. When any dropout rate was added to this number, a decision was made to evaluate a total of 40 patients.

Statistical analyses were calculated using the “Statistical Package for Social Sciences” (SPSS) Version 21.0 (SPSS inc., Chicago, IL, USA) program. *p* < 0.05 was accepted as statistically significant. The “Shapiro–Wilk” test was used to determine whether the data in the study were normally distributed. The demographic information of the patients was compared using the “Mann–Whitney U-test”. For data showing normal distribution, the changes in pre-treatment and post-treatment values within the group were determined using the “Paired Sample *t*-test”. For data not showing normal distribution, the changes in pre-treatment and post-treatment values within the group were determined using the “Wilcoxon Signed-Rank test”.

Changes over time within the groups and group–time interactions for continuous variables were assessed with mixed two-way repeated measures analysis of variance. The classification of effect sizes (f) was determined by calculating partial eta squared (f = 0.10 (small effects), f = 0.25 (medium effects) and f = 0.40 (large effects)) [29].

## 3. Results

Forty patients were evaluated for inclusion in the study. Two patients did not meet the inclusion criteria. Thirty-eight patients received the allocated intervention. However, four patients dropped out in the follow-up period. A total of 34 patients (age 57.26 ± 7.963, 88.23% female) were finally included in the analysis among two groups (17/17) who completed the study as Group 1 and Group 2 (Figure 1). When the personal and sociodemographic characteristics of the groups were evaluated, there was no statistically significant difference between the groups. Before treatment, a significant difference was found between the groups only in terms of gender distribution (*p* = 0.036) (Table 1).

When an intragroup evaluation was made, an improvement was found in our primary scale WOMAC values in both groups after 4 weeks of treatment (*p* < 0.05). However, there was no statistically significant difference between the groups (groupxtime interactions) in terms of WOMAC values (*p* = 0.086, Table 2).

When an intragroup evaluation was performed, there was a significant improvement in VAS (rest/activity/night), PPT values, joint position sense and TUG values in both groups after 4 weeks of treatment (*p* < 0.05). However, there was no significant difference between the groups (groupxtime interactions) in terms of VAS (rest, activity, night), PPT values, joint position sense and TUG values (*p* > 0.05; Table 2 and Table 3).

There was no significant improvement in IPAQ values in both groups (*p* > 0.05). There was no statistically significant difference between the groups (group*time interactions) in terms of IPAQ values (*p* = 0.086; Table 2).

There was a statistically significant improvement in hip flexion/external rotation/internal rotation range of motion values (*p* < 0.05). There was a statistically significant improvement in knee flexion ROM values only in Group 1 and foot dorsiflexion values only in Group 2 (*p* < 0.05). There was no statistically significant improvement in knee extension and foot plantar flexion ROM values in either group (*p* = 0.217, *p* = 0.215). Only the increase in hip external rotation ROM values was statistically greater in Group 1 compared to Group 2 (*p* = 0.022; Table 4).

In the intragroup evaluation, there was a statistically significant improvement in hip extension/abduction/adduction, knee flexion/extension and foot dorsiflexion muscle strength values in both groups (*p* < 0.05). In the hip flexion and foot plantar flexion muscle strength values, there was a statistically significant improvement only in Group 2 (*p* = 0.023, *p* = 0.011). There was no statistically significant difference between the groups (group*time interactions) in terms of muscle strength values (*p* > 0.05; Table 5).

In the intragroup evaluation, there was a statistically significant improvement in the mean values of physical function, physical role difficulty, pain and general health scores from the subheadings of SF-36 in both groups (*p* < 0.05). After treatment, there was a significant improvement in the mean values of energy (vitality) and emotional health scores from the subheadings of SF-36 in Group 2, while there was a significant improvement in the mean values of the social function score in Group 1 (*p* = 0.030, *p* = 0.06, *p* = 0.033). However, no statistically significant difference was found between the groups (group*time interactions) in terms of all subparameter values of SF-36 (group*time interactions) (*p* > 0.05; Table 2).

When the patient satisfaction levels were examined, after treatment, 41.2% of the patients in Group 1 stated that they were much better; 52.9% stated that they were better; and 5.9% stated that they were the same. In Group 2, 76.5% stated that they were much better, and 23.5% stated that they were better. During and at the end of the study, no patient was exposed to any unintended effects or harmed.

## 4. Discussion

In our study, we investigated the effect of proprioceptive training in patients with knee OA at different radiological levels. We evaluated the effectiveness of proprioceptive exercises applied in addition to strengthening exercises in patients with Grade 1–2 and Grade 3–4 knee OA. Our hypothesis was that proprioceptive and strengthening exercises would be more effective in Grade 1–2 compared to Grade 3–4 knee OA in terms of function, pain, proprioception, ROM, muscle strength, physical performance, physical activity and quality of life. After treatment, improvements were observed in all parameters in both groups. Contrary to our hypothesis, although there were significant results in favor of Grade 1–2 in some values of hip external rotation ROM between the groups, no significant difference was found between the groups in other parameters.

When demographic characteristics were evaluated, there was a gender difference between the groups before treatment (*p* = 0.036). However, there was no difference between the groups in terms of pain, functionality, joint position sense, physical performance, range of motion, muscle strength and quality of life before treatment (*p* > 0.05). Therefore, we do not think that this difference before treatment created a difference in the parameters evaluated after treatment.

There was a statistically significant improvement in the WOMAC total score in both groups after treatment. No significant difference was observed between the groups after treatment. However, when examined in terms of ES, the improvement was greater in Group 2. We believe that this situation is due to the greater decrease in activity pain in Grade 3–4 after treatment. In the literature, in a study where strengthening exercises were given in addition to proprioceptive exercises, a 16.37-point improvement was observed in the WOMAC score after 4 weeks of treatment [28]. In our study, the WOMAC score was 15.25 in Grade 3–4 and 12.74 in Grade 1–2. Our results are similar to the literature. In the study conducted by Rogers et al., significant improvements were observed in the WOMAC total score and subparameters such as pain, tenderness and physical function in individuals who received proprioceptive exercises in addition to strengthening exercises after 4 weeks of treatment [30]. In another study, it was found that strengthening and proprioceptive exercises provided significant improvements in WOMAC physical function scores in patients with Grade 1–2 knee OA [31]. Our study results regarding WOMAC scores showed improvements similar to literature.

One of the most important causes of disability in patients with knee OA is pain [7]. Previously, pain in OA was thought to be caused by mechanical stress and wear and tears due to aging. However, a systematic review examined the relationship between the gut microbiome, tryptophan-derived metabolites and osteoarthritis-related pain [32]. Two studies in the review noted a relationship between different tryptophan metabolites and pain levels in patients with OA [33,34]. Two other studies reported an association between lipopolysaccharide levels and pain in OA [35,36]. In conclusion, there is a potential link between gut microbiome dysbiosis and OA-associated pain. Given the increasing recognition of the gut–joint axis, proprioceptive training helps in reducing patient pain by interacting with systemic inflammation and metabolic pathways through proprioceptors.

No statistically significant difference was observed in all VAS values between the groups after treatment. However, when we also consider ES, the improvement in VAS activity and rest values in Grade 1–2 is greater. This table shows that proprioceptive work plays a more active role in reducing pain in the initial stages of the disease. In the study conducted by Kumar et al., the pain values of patients who received proprioceptive and strengthening exercises after 4 weeks of treatment were approximately halved after treatment [28]. In another study, it was determined that proprioceptive exercises given as 4-week home exercises significantly reduced pain [37]. The improvements in the pain values of the patients in our study are similar to the literature. Statistically significant improvements were observed in all PPT values in both groups after treatment. There was no significant difference in the increase in PPT values between the groups. However, Group 1 was superior to Group 2 in both heel and knee values in terms of ES. We believe that this situation is since the improvement in VAS values was superior in Group 1 in terms of ES. In a study conducted on patients diagnosed with knee OA, it was observed that proprioception exercises and strengthening exercises did not provide an improvement in PPT values after 4 weeks of treatment [10]. We attribute the effectiveness of the values in our study to the intense content of our proprioceptive exercise.

Since movement limitations in the knee joint can affect both the upper and lower joints, we evaluated the range of motion of the entire lower extremity in our study. In our study, a statistically significant increase was observed in hip flexion, hip external rotation and hip internal rotation values after treatment in both groups. However, the increase in hip external rotation angles between the groups was statistically superior in Group 1. We think that this may be since the decrease in patients’ pain was greater in terms of effect size. A significant improvement was observed in knee flexion angles only in Grade 1–2 and ankle dorsiflexion angles only in Grade 3–4. No statistically significant improvement was observed in knee extension and ankle plantar flexion ROM values in either group. We believe that this is since there were no limitations in the initial angles of the patients in these parameters. In a study examining the effects of proprioception exercises applied in addition to isometric knee exercises in patients with knee OA, a significant improvement was observed in knee ROM values after treatment [38]. Our study results are similar to the results of this study.

Patients with knee OA have poorer proprioception compared to individuals in the same age group [13]. In addition, studies have evaluated joint repositioning sensations at different knee angles (5°, 15°, 20°, 30°, 45°, 55°, 60°, 70°) between 0 and 90° [26,39]. However, it has been stated that evaluating knee joint position sense in medium angle positions (45–70°) is more effective [10]. After treatment, a statistically significant improvement was observed for joint position sense knee flexion (60° and 75°) values in both groups. However, no significant difference was found for all values between the groups after treatment. When we conducted an examination in terms of ES, higher EB was detected in Grade 1–2 compared to Grade 3–4. We think that this situation may be since proprioceptive exercises provided greater improvement in pain, PPT and ROM values in Grade 1–2. In one study, joint position sense was measured at 30°, 45° and 60° knee flexion angles. After 4 weeks of treatment, there was an improvement in EPH values for all angle values in both groups. The researchers found that the deviation in JPS value at 60° knee flexion angle was approximately halved in the proprioceptive group [28]. In our study, the deviation in joint position sense at 60° knee flexion angle decreased to one-fifth of the initial value in Grade 1–2 and less than half of the initial value in Grade 3–4. We attribute the greater improvement in joint position sense values in our study to the intensity and comprehensiveness of our exercise content.

Weak quadriceps muscle is a risk factor for OA, and the strength of this muscle is very important for recovery [10]. In addition, having strong hip muscles increases the success of the treatment [12]. In our study, a statistically significant improvement was observed in both groups in hip extension, hip abduction, hip adduction, knee flexion, knee extension and foot dorsiflexion muscle strength values. In hip flexion and ankle dorsiflexion muscle strength values, significant improvement was observed only in Grade 3–4. No significant difference was observed in muscle strength values after treatment between the groups. We attribute this situation to the fact that both groups received a common strengthening exercise program. In a study comparing proprioception and strengthening exercises with a control group, lower extremity knee extension, knee flexion, foot dorsiflexion and plantar flexion muscle strengths were measured, and the total value was stated. An increase was found in the total lower extremity muscle strength value in the experimental group after treatment [40]. Our results are similar to literature.

OARSI recommends the use of the TUG test to evaluate physical performance in patients with knee OA [23]. According to the TUG test used in our study, statistically significant improvements were noted in both groups after treatment. However, no significant difference was found between the groups after treatment. When we performed an evaluation in terms of ES, Grade 1–2 values were higher. We think that this situation is because Grade 1–2 patients showed a more substantial improvement in terms of VAS activity and PPT values. In another study, significant improvements were noted in the TUG test after treatment in a group administered strengthening exercises in addition to proprioceptive exercises, including kinesthesia, balance and agility exercises [30]. Although the walking distances applied in the test in our study were different, our results are similar to the literature.

The decrease in functionality in patients with knee OA affects the physical activity level of the patients. Therefore, we used the short version of IPAQ to determine the physical activity level of the patients in our study. No significant improvement was observed in IPAQ values in either group after treatment. We believe that this may be due to the short duration of our treatment and the fact that the patients’ pain did not completely disappear.

The symptoms of patients with knee OA, such as pain, joint limitation and deterioration of functional status, reduce the quality of life of patients. In our study, the SF-36 scale was used to evaluate the quality of life of patients, and after treatment, improvements were observed in the physical function, physical role difficulty, pain and general health subparameters in both groups. Significant improvements were observed in Grade 3–4 in emotional role difficulty, emotional health and energy (vitality) subparameters, and in Grade 1–2, in the social function subparameter. We attribute this difference in improvement between the groups to the fact that different groups showed more substantial improvement in different parameters. No significant difference was found between the groups for all SF-36 subparameters after treatment. We think that this situation is due to the lack of differences between the groups in terms of pain and functionality. In a study conducted on patients with Grade 1 and Grade 2 knee OA in the literature, proprioception and strengthening exercises were found to provide significant improvements in SF-36 energy (vitality) values [31]. In our study, no significant improvement was observed in SF-36 energy (vitality) values in Grade 1–2 patients. We think that this situation may be due to our shorter treatment period. In another study conducted by the same researchers with proprioception and strengthening exercises in patients with Grade 1 and Grade 2 knee OA, significant improvement was observed in SF-36 physical function, physical role difficulty and energy (vitality) subparameters after 8 weeks of treatment [13]. Similarly, in our study, a statistically significant improvement was found in SF-36 physical function and physical role difficulty subparameters. However, we think that the difference in the SF-36 energy (vitality) subparameter may be due to the difference in the treatment period.

Determining the level of satisfaction in patients is very important for treatment. In our study, the satisfaction level of patients was evaluated with GRS. According to the scale, 41.2% of patients in Grade 1–2 stated that they were much better; 52.9% stated that they were better; and 5.9% stated that they were the same. Meanwhile, 76.5% of patients in Grade 3–4 stated that they were much better, and 23.5% stated that they were better. There is only one study in the literature that measured the level of patient satisfaction with proprioceptive work. In this study, the patient satisfaction level was measured after 8 weeks of treatment, and it was determined that the satisfaction level of patients gradually increased at 6-month and 1-year follow-ups [41]. In our study, similar to the literature, the majority of patients stated that they were satisfied with the treatment.

According to our study results, differences between the groups were only noted in terms of hip external rotation values after treatment. This difference favored Group 1 (Grade 1–2). We think that the reason for the difference between the groups in only one parameter may be due to the small sample size or the short follow-up period. A larger number of patients or a longer follow-up of the patients may result in a greater difference between the two groups.

This study has several strengths. Our study is the first study in the literature to evaluate the effectiveness of proprioceptive training in patients with knee OA according to radiological stages. There is no standard protocol in the literature regarding proprioception exercises used in patients with knee OA. It can be stated that our study is superior to other studies in terms of the number and content of exercises used in literature. Our study is the first study in the field to employ telerehabilitation method.

Our study has some limitations. The short-term effectiveness of proprioceptive training in patients with knee OA was evaluated according to radiological levels. However, its long-term effect could not be evaluated. The small sample size is another limitation of our study. The lack of a control group to better evaluate the effectiveness of the treatment is another limitation. In the telerehabilitation method, patients performed the exercises simultaneously with the therapist via video call via WhatsApp. This method is an innovative application that facilitates the monitoring of patients, facilitates their compliance with the exercise and saves transportation costs and time. However, the lack of standardization in terms of the environment in which the patients are located may be a limitation.

## 5. Conclusions

For physicians and physiotherapists, proprioceptive training is an effective method that can be used safely in knee OA. Proprioceptive exercises applied in addition to strengthening exercises can help patients have a shorter recovery process and achieve more effective results. The results of our study showed that proprioceptive training was effective in terms of pain, pressure pain threshold, proprioceptive sensation, ROM, muscle strength, physical performance, functional status and quality of life, regardless of the radiological stage. It was also determined that proprioceptive training did not result in a significant difference in the parameters evaluated between Grades 1–2 and Grades 3–4. Therefore, knee OA can be included in a treatment program, regardless of its radiological degree. More studies are needed on this subject. Large population studies are needed to investigate the long-term effects of proprioceptive training. Future studies may include a group that comprises only strengthening exercises, so that the effects of proprioceptive exercises alone can be examined.

## Figures and Tables

**Table 1:** Demographic and clinical characteristics of the groups.

		Grade 1–2 (Group 1) Mean ± SD *n*%	Grade 3–4 (Group 2) Mean ± SD *n*%	*p*
Age (years)		55.35 ± 8.33	59.18 ± 7.08	0.262
BKI (kg/m^2^)		27.75 ± 4.67	30.04 ± 3.73	0.105
Gender	Woman	13 (%76.5)	17 (%100)	**0.036**
	Man	4 (%23.5)	0 (%0)	
Marital status	Married	15 (%88.2)	16 (%93.3)	0.529
	Single	1 (%5.9)	0 (%0)	
	Widow	1 (%5.9)	1 (%5.9)	
Occupation	Retired	4 (%23.5)	1 (%5.9)	0.059
	Officer	2 (%11.8)	1 (%5.9)	
	Private sector	1 (%5.9)	0 (%0)	
	No	0 (%0)	0 (%0)	
	Housewife	10 (%58.8)	15 (%88.2)	
Presence of crepitation	Yes	13 (%76.5)	16 (%94.10)	0.152
	No	4 (%23.5)	1 (%5.9)	
Affected side	Right	6 (%40)	9 (%60)	0.734
	Left	9 (%60)	6 (%40)	
Presence of comorbid disease	Yes	8 (%47.1)	9 (%52.9)	0.735
	No	9 (%52.9)	8 (%47.1)	
Radiological level	Grade 1	Grade 2	Grade 3	Grade 4
	3 (%8)	14 (%41.2)	11 (%32.4)	6 (%17.6)

(BMI: Body Mass Index, SD: Standard Deviation *p* < 0.05, bold text: significant finding).

**Table 2:** Comparison of WOMAC, TUG, IPAQ and SF-36 values within and between groups.

	Before Treatment Mean ± SD	After Treatment Mean ± SD	Intragroup Difference Analysis		Within Group Mean Change [%95 CI] (Min–Max)	ES	Intergroup Difference Analysis			
			*p* ^1^	T/Z			Time *p*^2^		TimexGroup *p*^2^	
WOMAC										
Group 1	33.16 ± 19.48	20.42 ± 16.71	**0.001**	4.02	−12.74 (6.03–19.44)	0.7	0.521	(0.013)	0.086	(0.089)
Group 2	42.82 ± 18.32	27.57 ± 13.71	**0**	5.68	−15.25 (9.56–20.53)	0.94				
TUG *										
Group 1	10.18 ± 2.81 (6.18–16.76)	8.21 ± 1.46 (5.84–11.58)	**0**	Z = −3.62	−1.97	0.87	**<0.001**	(0.634)	0.805	(0.002)
Group 2	11.74 ± 3.89 (7.56–23)	9.89 ± 3.24 (6.56–18.81)	**0**	Z = −3.62	−1.85	0.51				
IPAQ *										
Group 1	2059.05 ± 2329.43	3295.58 ± 4137.40	0.149	Z = −1.44	1236.53	0.36	0.521	(0.013)	0.086	(0.089)
Group 2	2762.97 ± 5193.37	2189.05 ± 4014.46	0.332	Z= −0.97	−573.92	0.12				
SF-36 Physical functioning										
Group 1	53.23 ± 22.70	69.11 ± 19.78	**0.001**	−3.92	15.88 (−24.45 + 7.29)	0.74	**<0.001**	(0.489)	0.534	(0.012)
Group 2	42.35 ± 16.01	55 ± 20.61	**0.001**	−3.97	12.65 (−19.39 + 5.89)	1.48				
SF-36 Role limitations due to physical health										
Group 1	32.35 ± 38.28	67.64 ± 43.08	**0.004**	−3.35	35.29 (−57.58 + 13)	0.86	**<0.001**	(0.401)	0.660	(0.006)
Group 2	23.52 ± 34.76	52.64 ± 38.45	**0.006**	−3.19	29.12 (−48.44 + 9.78)	1.12				
SF-36 Role limitations due to emotional problems										
Group 1	45.08 ± 38.98	66.64 ± 35.38	0.086	−1.83	21.56 (−46.51–3.39)	0.57	**0.013**	(0.179)	0.795	(0.002)
Group 2	33.32 ± 31.18	50.98 ± 42.68	0.07	−1.94	17.66 (−48.44 + 9.78)	0.47				
SF-36 Energy/fatigue										
Group 1	47.94 ± 22.50	54.70 ± 21.17	0.177	−1.41	6.76 (−16.92–3.39)	0.3	**0.013**	(0.179)	0.588	(0.009)
Group 2	44.11 ± 19.22	54.41 ± 19.19	**0.03**	−2.38	10.3 (−36.93–161)	0.53				
SF-36 Emotional well-being										
Group 1	62.58 ± 12.87	62.29 ± 17.39	0.131	−1.59	−3.29 (−10.97–1.55)	0.01	**0.001**	(0.274)	0.131	(0.070)
Group 2	56.00 ± 17.72	68.26 ± 18.35	**0.006**	−3.15	12.26 (−20.50 + 4.02)	0.67				
SF-36 Social functioning										
Group 1	76.47 ± 21.59	86.02 ± 17.04	**0.033**	−2.34	9.62 (−18.22 + 0.89)	0.49	**0.124**	(0.149)	0.483	(0.016)
Group 2	72.05 ± 27.43	77.20 ± 29.39	0.288	−1.1	5.15 (−15.06–4.77)	0.18				
SF-36 Pain										
Group 1	54.11 ± 29.18	66.79 ± 19.08	**0.016**	−2.7	12.68 (−22.62 + 2.72)	0.51	**<0.001**	0.482	0.084	(0.090)
Group 2	39.11 ± 22.39	64.11 ± 19.82	**0**	−4.92	25 (−35.75 + 14.24)	1.18				
SF-36 General health										
Group 1	47.05 ± 25.55	62.05 ± 21.55	**0.004**	−3.33	15 (−24.53 + 5.46)	0.63	**<0.001**	0.328	0.145	0.065
Group 2	48.52 ± 16.46	55.29 ± 18.24	**0.049**	−2.12	6.77 (−13.50 + 0.02)	0.38				

Note: (*p*^1^: Paired Sample *t*-test, *p*^2^: Two-way repeated measures analysis of variance with a mixed model. Figures in parentheses are effect sizes. *: Wilcoxon Signed-Rank test) (Group 1: Grade 1–2, Group 2: Grade 3–4, WOMAC: Western Ontario and McMaster Universities Arthritis, TUG: Timed Up and Go Test, IPAQ: International Physical Activity Questionnaire, SD: Standard Deviation, ES: Effect Size, CI: Confidence Interval, bold text: significant finding, *p* < 0.05).

**Table 3:** Comparison of pain intensity and joint position sense values within and between groups.

	Before Treatment Mean ± SD	After Treatment Mean ± SD	Intragroup Difference Analysis		Within Group Mean Change [%95 CI] (Min–Max)	ES	Intergroup Difference Analysis			
			*p* ^1^	T			Time		TimexGroup	
							*p* ^2^		*p* ^2^	
VAS rest cm										
Group 1	3.53 ± 2.85	0.76 ± 1.39	**0.001**	4.17	−2.77 (1.36–4.16)	1.23	**<0.001**	(0.459)	0.585	(0.09)
Group 2	3.41 ± 3.64	1.18 ± 2.27	**0.005**	3.22	−2.23 (0.76–3.70)	0.73				
VAS activity cm										
Group 1	5.71 ± 2.99	2.53 ± 2.19	**0**	5.44	−3.18 (1.94–4.41)	1.21	**<0.001**	(0.679)	0.227	(0.045)
Group 2	6.88 ± 3.19	2.59 ± 2.34	**0**	6.17	−4.29 (2.82–5.76)	1.53				
VAS night cm										
Group 1	4.88 ± 4.37	2.06 ± 2.92	**0.001**	4.31	−2.82 (1.43–4.21)	0.75	**<0.001**	(0.486)	0.953	(<0.001)
Group 2	5.06 ± 4.16	2.29 ± 2.80	**0.002**	3.68	−2.77 (1.17–4.35)	0.78				
PPT medial point of knee										
Group 1	4.64 ± 1.07	6.24 ± 1.05	**0**	−7.46	1.6 (−2.05 + 1.14)	1.5	**<0.001**	(0.712)	0.805	(0.002)
Group 2	3.96 ± 1.46	5.47 ± 1.71	**0**	−5.46	1.51 (−2.09 + 0.92)	0.94				
PPT medial point of heel										
Group 1	7.70 ± 2.17	10.05 ± 1.63	**0**	−6.23	2.35 (−3.14 + 1.54)	1.22	**<0.001**	(0.677)	0.524	(0.013)
Group 2	7.05 ± 2.92	9.80 ± 2.46	**0**	−5.55	2.75 (−3.79 + 1.69)	1.01				
(30° active joint position sense)										
Group 1	2.21 ± 2.07	0.40 ± 0.39	**0.002**	3.64	−1.81 (0.75–2.85)	1.21	**<0.001**	(0.554)	0.701	(0.005)
Group 2	3.87 ± 3.19	1.84 ± 2.66	**0**	5.71	−2.03 (1.28–2.79)	0.69				
(15° active joint position sense)										
Group 1	2.58 ± 2.61	0.50 ± 0.56	**0.002**	3.74	−2.08 (0.90–3.25)	1.1	**<0.001**	(0.394)	0.500	(0.014)
Group 2	2.70 ± 2.73	1.16 ± 1.81	**0.015**	2.72	−1.54 (0.33–2.73)	0.66				

Notes: (*p*^1^: Paired Sample *t*-test, *p*^2^: Two-way repeated measures analysis of variance with a mixed model. Figures in parentheses are effect sizes.) (Group 1: Grade 1–2, Group 2: Grade 3–4, VAS: Visual Analog Scale, PPT: Pressure Pain Threshold, SD: Standard Deviation, ES: Effect Size, CI: Confidence Interval, bold text: significant finding, *p* < 0.05).

**Table 4:** Comparison of hip, knee and ankle joint range of motion within and between groups.

	Before Treatment Mean ± SD	After Treatment Mean ± SD	Intragroup Difference Analysis		Within Group Mean Change [%95 CI] (Min–Max)	ES	Intergroup Difference Analysis			
			*p* ^1^	T/Z			Time		TimexGroup	
							*p* ^2^		*p* ^2^	
Hip Flexion (°)										
Group 1	106.71 ± 17.37	116.88 ± 12.68	**0**	−4.51	10.17 (−14.95 + 5.39)	0.66	**<0.001**	(0.5349)	0.584	(0.009)
Group 2	97.71 ± 16.68	106.18 ± 15.05	**0.001**	−4.03	8.47 (−12.91 + 4.02)	0.53				
Hip ER (°)										
Group 1	32.41 ± 7.34	42.00 ± 3.33	**0**	−6.28	9.59 (−12.82 + 6.35)	1.66	**<0.001**	(0.632)	**0.022**	(0.154)
Group 2	35.12 ± 7.17	40.00 ± 7.28	**0.001**	−4	4.88 (−7.46 + 2.30)	0.67				
Hip IR (°)										
Group 1	36.71 ±6.98	42.94 ± 3.09	**0.001**	−3.85	6.23 (−9.66 + 2.80)	1.15	**<0.001**	(0.405)	0.120	(0.074)
Group 2	37.82 ± 8.17	40.88 ± 4.41	**0.018**	−2.64	3.06 (−5.51 + 0.60)	0.46				
Knee Flexion (°)										
Group 1	112.47 ± 13.38	119.65 ± 11.55	**0.003**	−3.48	7.18 (−11.54 + 2.80)	0.57	**0.001**	(0.293)	0.826	(0.002)
Group 2)	109.12 ± 15.63	115.47 ± 13.20	0.057	−2.05	6.35 (−12.90–0.19)	0.43				
* Knee Extension (°)										
Group 1	0.00 ± 0.00	0.00 ± 0.00	1	Z = 0.00	0	0	0.217	(0.047)	0.863	(0.001)
Group 2	−3.82 ± 9.10	−0.29 ± 1.21	0.109	Z = 0.00	3.53	0.54				
* Plantar Flexion (°)										
Group 1	45.00 ± 0.00	45.00 ± 0.00	1	Z = 0.00	0	0	0.215	(0.048)	0.532	(0.012)
Group 2	44.12 ± 3.63	45.00 ± 0.00	0.317	Z = −1.00	0.88	0.34				
Dorsiflexion (°)										
Group 1	18.82 ± 2.18	19.41 ± 1.66	0.163	−1.46	0.59(−1.44–0.26)	0.3	**0.013**	(0.179)	0.114	(0.076)
Group 2	16.06 ± 5.21	18.53 ± 3.43	**0.037**	−2.27	2.47(−4.77–0.17)	0.86				

Notes: (*p*^1^: Paired Sample *t*-test, *p*^2^: Two-way repeated measures analysis of variance with a mixed model. Figures in parentheses are effect sizes. *: Wilcoxon Signed-Rank test) (Group 1: Grade 1–2, Group 2: Grade 3–4, ER: External Rotation, IR: Internal Rotation, SD: Standard Deviation, ES: Effect Size, CI: Confidence Interval, bold text: signicant finding, *p* < 0.05).

**Table 5:** Comparison of hip, knee and ankle muscle strength values within and between groups.

	Before Treatment Mean ± SD	After Treatment Mean ± SD	Intragroup Difference Analysis		Within Group Mean Change [%95 CI] (Min–Max)	ES	Intergroup Difference Analysis			
			*p* ^1^	T/Z			Time		TimexGroup	
							*p* ^2^		*p* ^2^	
Hip Flexion (°)										
Group 1	106.71 ± 17.37	116.88 ± 12.68	**0**	−4.51	10.17 (−14.95 + 5.39)	0.66	**<0.001**	(0.5349)	0.584	(0.009)
Group 2	97.71 ± 16.68	106.18 ± 15.05	**0.001**	−4.03	8.47 (−12.91 + 4.02)	0.53				
Hip ER (°)										
Group 1	32.41 ± 7.34	42.00 ± 3.33	**0**	−6.28	9.59 (−12.82 + 6.35)	1.66	**<0.001**	(0.632)	**0.022**	(0.154)
Group 2	35.12 ± 7.17	40.00 ± 7.28	**0.001**	−4	4.88 (−7.46 + 2.30)	0.67				
Hip IR (°)										
Group 1	36.71 ±6.98	42.94 ± 3.09	**0.001**	−3.85	6.23 (−9.66 + 2.80)	1.15	**<0.001**	(0.405)	0.120	(0.074)
Group 2	37.82 ± 8.17	40.88 ± 4.41	**0.018**	−2.64	3.06 (−5.51 + 0.60)	0.46				
Knee Flexion (°)										
Group 1	112.47 ± 13.38	119.65 ± 11.55	**0.003**	−3.48	7.18 (−11.54 + 2.80)	0.57	**0.001**	(0.293)	0.826	(0.002)
Group 2)	109.12 ± 15.63	115.47 ± 13.20	0.057	−2.05	6.35 (−12.90–0.19)	0.43				
* Knee Extension (°)										
Group 1	0.00 ± 0.00	0.00 ± 0.00	1	Z = 0.00	0	0	0.217	(0.047)	0.863	(0.001)
Group 2	−3.82 ± 9.10	−0.29 ± 1.21	0.109	Z = 0.00	3.53	0.54				
* Plantar Flexion (°)										
Group 1	45.00 ± 0.00	45.00 ± 0.00	1	Z = 0.00	0	0	0.215	(0.048)	0.532	(0.012)
Group 2	44.12 ± 3.63	45.00 ± 0.00	0.317	Z = −1.00	0.88	0.34				
Dorsiflexion (°)										
Group 1	18.82 ± 2.18	19.41 ± 1.66	0.163	−1.46	0.59 (−1.44–0.26)	0.3	**0.013**	(0.179)	0.114	(0.076)
Group 2	16.06 ± 5.21	18.53 ± 3.43	**0.037**	−2.27	2.47 (−4.77–0.17)	0.86				

Notes: (*p*^1^: Paired Sample *t*-test, *p*^2^*:* Two-way repeated measures analysis of variance with a mixed model. Figures in parentheses are effect sizes. *: Wilcoxon Signed-Rank test) (Group 1: Grade 1–2, Group 2: Grade 3–4, ER: External Rotation, IR: Internal Rotation, SD: Standard Deviation, ES: Effect Size, CI: Confidence Interval, bold text: significant finding, *p* < 0.05).

## Data Availability

All authors give permission for the data in the article to be published in MDPI journals.

## Abbreviations

The following abbreviations are used in this manuscript:

OA	Osteoarthritis
WOMAC	Western Ontario and McMaster Universities Arthritis
NSAIDs	Non-Steroidal Anti-Inflammatory Drugs
ACR	American College of Rheumatology
GRS	Global Range Scale
VAS	Visual Analog Scale
PPT	Pressure Pain Threshold
JPS	Joint Position Sense
ROM	Range of Motion
IPAQ	International Physical Activity Questionnaire
TUG	Timed Up and Go Test
ES	Effect Size

## Appendix A

### Proprioceptive Training Progression

Week 1

➢ Proprioceptive exercises (15 steps)
➢ Balance exercises
  - Weight transfer exercises on one leg with eyes open forward, side and back (10 s)
➢ Coordination exercises
  - Bilateral knee flexion–extension with eyes open in sitting position (10 repetitions)
  - Heel sliding exercises with eyes open in sitting position (10 repetitions)
➢ Strengthening exercises
  - 5 min warm-up (light jogging)
  - Hip abduction, adduction, flexion and extension exercises while lying on the back (10 repetitions)
  - Knee extension and flexion exercises in sitting position (10 repetitions)
  - Squat in standing position leaning on the wall (8 repetitions)

Week 2

➢ Proprioceptive exercises (20 steps)
➢ Balance exercises
  - Weight transfer exercises forward, side and back while standing on one leg and eyes open)
➢ Coordination exercises
  - Bilateral knee flexion–extension in sitting position with eyes open (15 repetitions)
  - Heel driving exercises in sitting position with eyes open (15 repetitions)
➢ Strengthening exercises
  - 5 min warm-up (light jogging)
  - Hip abduction, adduction, flexion and extension exercises while lying on the back (0.5 kg, 10 repetitions)
  - Knee extension and flexion exercises while sitting (0.5 kg, 10 repetitions)
  - Squat in standing position leaning on the wall (10 repetitions)

Week 3

➢ Proprioceptive exercises (25 steps)
➢ Balance exercises
  - Weight transfer exercises on one leg with eyes closed, forward, side and back (10 s)
➢ Coordination exercises
  - Bilateral knee flexion–extension in sitting position with eyes closed (10 repetitions)
  - Heel driving in sitting position with eyes closed (10 repetitions)
➢ Strengthening exercises
  - 5 min warm-up (light jogging)
  - Hip abduction, adduction, flexion and extension exercises while lying on the back (1 kg, 10 repetitions)
  - Knee extension and flexion exercises while sitting (1 kg, 10 repetitions)
  - Squat in standing position leaning on the wall (12 repetitions)

Week 4

➢ Proprioceptive exercises (30 steps)
➢ Balance exercises
  - Weight transfer exercises on one leg with eyes closed, forward, side and back (15 s)
➢ Coordination exercises
  - Bilateral knee flexion–extension with eyes closed while sitting (15 repetitions)
  - Heel driving exercises with eyes closed while sitting (15 repetitions)
➢ Strengthening exercises
  - 5 min warm-up (light jogging)
  - Hip abduction, adduction, flexion and extension exercises while lying on the back (1.5 kg, 10 repetitions)
  - Knee extension and flexion exercises while sitting (1.5 kg, 10 repetitions)
  - Standing squat in supine position against a wall (15 repetitions)
